# Increasing venous thromboembolism risk assessment through a whole hospital-based intervention: a pre-post service evaluation to demonstrate quality improvement

**DOI:** 10.1093/intqhc/mzae019

**Published:** 2024-03-01

**Authors:** Juliana Abboud, Niaz Shaikh, Musthafa Moosa, Martin Dempster, Pauline Adair

**Affiliations:** Centre for Improving Health Related Quality of Life, School of Psychology, Queens University Belfast, David Keir Building, 18-30 Malone Road, Belfast BT9 5BN, United Kingdom; Rashid Hospital, Dubai Academic Health Corporation, Umm Hurair II 315, PO Box 7272, Dubai, United Arab Emirates; Rashid Hospital, Dubai Academic Health Corporation, Umm Hurair II 315, PO Box 7272, Dubai, United Arab Emirates; Rashid Hospital, Dubai Academic Health Corporation, Umm Hurair II 315, PO Box 7272, Dubai, United Arab Emirates; Centre for Improving Health Related Quality of Life, School of Psychology, Queens University Belfast, David Keir Building, 18-30 Malone Road, Belfast BT9 5BN, United Kingdom; Centre for Improving Health Related Quality of Life, School of Psychology, Queens University Belfast, David Keir Building, 18-30 Malone Road, Belfast BT9 5BN, United Kingdom

**Keywords:** quality improvement, venous thromboembolism, VTE risk assessment, patient safety

## Abstract

Venous thromboembolism (VTE) is a primary cause of morbidity and mortality in hospitalized patients. VTE risk assessment is a crucial part of the VTE prevention guideline. However, VTE risk assessment was not consistently undertaken for admitted patients. The aim of this study was to identify whether a quality improvement project implemented to change documentation of VTE risk assessment for hospitalized patients impacted patient safety by decreasing the rate of VTE incidences. The study was set in a 600+ bed acute hospital that provides medical and surgical services for adult patients during the period October 2018–September 2020. The hospital adopted the American College of Chest Physicians (ACCP) 9th edition VTE prevention guidelines and followed the Modified Caprini risk assessment tool. Following the FOCUS-Plan-Do-Check-Act (FOCUS PDCA) improvement methodology, the improvement team implemented multicomponent interventions over a 3-month period, including conducting educational sessions, sharing VTE documentation compliance results, giving reminders during rounds, assigning a VTE liaison physician within each clinical specialty, and updating and communicating the hospital adopted VTE guidelines. A total of 17 612 patients were included, respectively, 8971 in pre-intervention and 8641 post-intervention period. Documentation of VTE risk assessment upon admission increased significantly in the post quality improvement intervention period (60% vs. 42%, relative increase of 30%, χ2 = 1.43, *P* < 0.001). The run chart trend analysis demonstrated significant improvement shift and improvement trend after quality improvement project implementation, and it was sustained for 15 months. There was no impact on patient safety with a slight not statistically significant decrease in the VTE incidences rate post intervention period (0.4% vs. 0.5%, relative decrease of 1%, χ2 = 0.82, *P* < 0.397). The quality improvement project intervention significantly increased the percentage of patients assessed for VTE risk in a hospital setting.

## Introduction

Venous thromboembolism (VTE) is a primary cause of morbidity and mortality in hospitalized patients. Once a patient develops a VTE event, there is a considerable risk of developing into one of the more life-threatening deep vein thrombosis, pulmonary embolism [1]. VTE can be prevented in hospitalized patients [2–4]. Evidence-based clinical practice guidelines followed by hospitals outlined recommendations to prevent venous thromboembolism in hospitalized patients [5–7]. An effective VTE prevention guideline consists of a VTE risk assessment, a risk of bleeding assessment, and clinical decision-making on prophylactic choices based on the combination of VTE and bleeding risk factors. VTE risk assessment is a crucial part of the VTE guideline. Several studies noted that VTE risk assessment was not consistently done for admitted patients, and thus appropriate prophylaxis was not always received [8–10].

At our organization, a performance compliance gap was identified when data were examined over 6 months for patients’ records admitted during the period October 2018–March 2019. The results revealed that the VTE risk assessment tool was documented for only 42% of the admitted patients during that period, denoting a gap between clinical practice and the recommendations of the adopted guidelines in the hospital setting.

As such, an improvement team was assigned by hospital leadership to improve the VTE risk assessment practice. The team used the FOCUS-Plan-Do-Check-Act (FOCUS-PDCA) structural improvement method [11]. It was expected that the interventions implemented through the quality improvement project would increase the adherence to VTE risk assessment practice by admitting physicians and documenting it in the electrical medical record.

The aim of the study was to identify whether the implemented quality improvement project has increased documentation of VTE risk assessment upon admission and within 24 hours and impacted patient safety by decreasing the rate of VTE incidences that occurred during hospitalization or within 28 days post discharge.

## Methods

This study focuses on quality improvement to investigate changes in VTE risk assessment practice after the implementation of a quality improvement project. It is reported based on the Standards for Quality Improvement Reporting Excellence (SQUIRE 2.0) guidelines [12] checklist (See Supplementary Material 1).

## Setting

This project was set in a 600+ bed acute hospital that provides medical and surgical services for adult patients. The designated hospital had an Electronic Medical Record that achieved a score ‘stage 6’ based on HIMSS Analytics Electronic Medical Record Adoption Model (EMRAM). EMRAM is an eight-stage model (0–7), zero (least mature) to seven (most mature); the maturity will increase by achieving a paperless medical record and relying on automation in clinical decision support to support the optimization of patient care [13].

The hospital adopted the American College of Chest Physicians (ACCP) 9th edition VTE prevention guidelines [6] and followed the ‘Modified Caprini risk assessment tool’ [14] to assess the VTE risk. The VTE risk assessment tool was integrated into the hospital electronic medical record system in July 2018 as per a previous quality improvement project undertaken by the improvement team. The tool is a combination of factors identified during the initial patient assessment that adds to a total score suggesting a patient’s vulnerability or risk of developing VTE. Accordingly, physicians decide the appropriate prophylaxis therapy for each patient’s risk profile.

### Intervention(s)

The VTE improvement team followed the FOCUS PDCA method through implementing the following steps:

#### Find an opportunity

The VTE improvement team defined the project aim statement as ‘Ensure that over 80% of the inpatients are assessed for VTE risk upon admission by December 2019’.

#### Organize a team

The hospital administration formed a multidisciplinary VTE team. The project team included physicians from different clinical specialities and representatives from the quality department. Physicians were selected due to their involvement in the VTE risk assessment process. The team lead was a senior physician responsible for overseeing the project implementation and quality members to facilitate the FOCUS PDCA process and analyse data. Team members were responsible for planning, implementing, and monitoring the interventions within their respective departments.

#### Clarify the process

The project team members explored the current VTE guidelines practice and described it using a process flow chart. The following steps were followed on admission by the admitting doctor:

- Conduct patient assessment.
- Assess patients for risk of VTE using the electronic tool and identify the risk score.
- Assess patients for bleeding risk.
- Evaluate the risks and benefits of prescribing VTE prophylaxis.
- Prescribe Pharmacological or Mechanical prophylaxis if indicated.

Moreover, VTE risk assessment results of data collected in 2018 were scrutinized and used as baseline data.

#### Understand the process

The project team conducted a brainstorming session to gather feedback from the team members about their perceptions of why the VTE risk assessment tool was not completed in their respective departments. The quality team member facilitated the discussion to ensure all opinions were captured. The following barriers were identified based on the discussion:

- VTE prophylaxis (first dose) was administered without formal VTE risk assessment.
- Lack of awareness of the admitting physicians that VTE risk assessment tool should be completed upon admission.
- There was no formal monitoring of physicians’ performance in completing the VTE form.
- The VTE tool was not part of the admission bundle.

#### Select the process improvement

The improvement team selected the intervention components followed in the quality improvement project based on the best practices found to be effective in other hospital settings and the published literature [15, 16]. The interventions to address the improvement opportunities included [1]: conducting educational sessions [2], sharing VTE documentation compliance results [3], giving reminders during rounds [4], assigning a VTE liaison physician within each clinical specialty, and [5] updating and communicating the hospital adopted VTE guidelines.

#### Plan-Do-Check-Act

The PDCA cycles presented in Table 1 were implemented simultaneously over a 3-month period, April to June 2019, targeting all physicians from medical and surgical specialties. They included education about the VTE guidelines components and the importance of implementation; assigning a VTE liaison physician in each clinical department; sharing the results of the VTE risk assessment compliance with the different specialties during their staff meetings by the assigned VTE liaison physician. Reminders by the senior physicians were communicated to admitting physicians during daily rounds. The hospital VTE prevention clinical practices guidelines were updated to include VTE risk assessment to be completed on admission by the admitting physicians. Two recommendations were raised by the VTE team to hospital leadership to include the VTE risk assessment tool in the admission bundle and the documentation compliance to be part of the physicians’ performance evaluation.

**Table 1.** Implementation of PDCA.

Cycle	Plan	Do	Check	Act
1	**VTE liaison physician**: Assign a VTE liaison physician in each clinical speciality	The VTE liaison physicians were assigned at the beginning of the project and were responsible for conducting the education sessions and following up the documentation compliance	Formation of the VTE liaison team, including a member from each department and unit in March 2019	VTE liaison team was active in reviewing the performance of their departments after the implementation of the quality improvement project
2	**Conducting educational sessions**: A 1-hour teaching session was designed to cover the VTE guidelines components and the importance of implementation	A total of 18 educational sessions were conducted during the 2 months period to cover targeted physicians	Percentage documentation of the VTE risk assessment monitored monthly	Periodic educational sessions were planned to be continuously conducted mainly for new physicians and residents
3	**Sharing compliance results**: Share the results of the VTE risk assessment compliance with the physicians	VTE compliance results per department were presented to physicians during the clinical head of departments meetings and speciality staff meetings by the VTE liaison physicians	Percentage documentation of the VTE risk assessment monitored monthly	Quarterly statistics VTE compliance results were planned to be communicated continuously to physicians by conducting presentations or by sending emails
4	**Reminders** Give reminders during ward rounds	Reminders were emphasized by the senior physicians to the admitting physicians during the daily rounds about completing the VTE risk assessment on admission	Percentage documentation of the VTE risk assessment monitored monthly	Quarterly statistics VTE compliance results were planned to be communicated continuously to physicians by conducting presentations or by sending emails
5	**Hospital adopted VTE guidelines**: Update and communicate the hospital adopted VTE guidelines to standardize the practice	VTE prevention Clinical practices guidelines were updated to include VTE Risk assessment to be completed upon admission by the admitting physicians and communicated to all staff through the internal announcement system	The policy was updated and approved by the hospital CEO	VTE Clinical Practice guidelines were planned to be reviewed at least every 2 years or when new evidence warrants modifications of recommendations or upon change of professional consensus as per hospital policy

## Study of the intervention(s)

Retrospective data extracted from the electronic database, including all adult patients aged 18 years or older who were admitted to the hospital with an overnight stay greater than 24 hours during the study period between October 2018 and March 2019 (Pre-intervention) and July to December 2019 (Post-intervention), were analysed using the Statistical Package for the Social Sciences (SPSS) version 26.

## Measures

The primary outcome measure was the percentage of patients for whom a risk assessment was documented upon admission as per the VTE guidelines. The secondary outcome measures were the percentage of patients for whom a risk assessment was documented within 24 hours, within hospital stay with no time limit, and the VTE incidences rate during patient hospitalization or within 28 days post discharge.

## Data analysis

The outcome measures about the documentation of the VTE risk assessment were analysed and compared between the two time periods before and after project implementation by using the chi-squared test [17]. The 95% Confidence Interval for the overall change in percentage compliance was calculated by the ‘modified Wald method’.

To visualize variation within the data and identify an improvement over time, run charts of time-series data displayed in graphs were used to understand if the interventions made lead to improvement. Improvement shifts were analysed taking into consideration the following probability-based rule: an improvement was reported if six or more consecutive data points were all above the median and data points that fell on the median were not counted since they did not make or break a trend. An initial median using baseline data was calculated for the time period (October 2018–March 2019). To identify a positive trend of improvement, the rule five or more consecutive points all going up was applied. If the value of two or more consecutive points were the same, the first point was only counted [18].

The VTE incidences per number of discharges based on the discharge diagnosis according to the tenth International Statistical Classifications of Diseases (ICD-10) [19] and the hospital morbidity review reports were calculated pre and post quality improvement project implementation.

## Results

### VTE risk assessment documentation after an improvement project

A total of 17 612 patients were admitted to the hospital during the study period and included in the analysis, 8971 patients during pre-intervention period and 8641 patients during post-intervention period. Demographic comparison data are presented in Table 2. There was strong evidence of an increase in the percentage of patients for whom a VTE risk assessment was documented after implementing the improvement project. Documentation of VTE risk assessment upon admission was improved from 42% (*n* = 3790) before implementation of the project to 60% (*n* = 5219) after implementation (relative increase of 30%) (*P* < 0.001). However, the project-targeted objective of reaching 80% VTE risk assessment documentation by December 2019 was not reached. On the other hand, documentation of VTE risk assessment within 24 hours was improved from 55% (*n* = 4989) before to 76% (*n* = 6513) after the project implementation (relative increase of 38%) (*P* < 0.001) and within hospital stay without time constraint from 71% (*n* = 6395) before to 88% (*n* = 7511) as presented in Table 3.

**Table 2.** Demographic data pre and post improvement project implementation.

Retrospective database evaluation data set	Pre intervention(*n* = 8971)	Post intervention(*n* = 8641)	*P* Value
**Age (years), Mean (SD)**	45 (18.03)	46 (18.01)	0.95
**Age group, no (%)**			
≤40 years	4115 (46%)	4084 (47%)	0.245
40–60 years	2794 (31%)	2646 (31%)	
61–74 years	1257 (14%)	1185 (14%)	
≥75 years	805 (9%)	726 (8%)	
**Gender, no (%)**			
Male	6233 (69%)	6056 (70%)	0.271
Female	2738 (31%)	2585 (30%)	
**Specialities, no (%)**			
Medical	4979 (55.5%)	4782 (55.3%)	0.831
Surgical	3992 (44.5%)	3859 (44.7%)	

**Table 3.** VTE risk assessment documentation pre and post project implementation.

VTE assessment documentation	Pre intervention (Oct 2018–Mar 2019)(*n* = 8971)	Post intervention (July–Dec 2019) (*n* = 8641)	Relative increase(95% CI)	*P* Value
On admission	3790 (42%)	5219 (60%)	1.43 (1.38–1.47)	0.001
Within 24 hours	4989 (55%)	6513 (76%)	1.36 (1.32–1.39)	0.001
Overall, during hospital stay, no time constraint	6395 (71%)	7511 (88%)	1.21 (1.20–1.24)	0.001

**Abbreviation**: *n* = total number of participants in the study; CI = Confidence Interval.

Note: *P*-Value ≤ 0.05 is statistically significant.

### VTE risk assessment documentation over time

**Figure 1:**
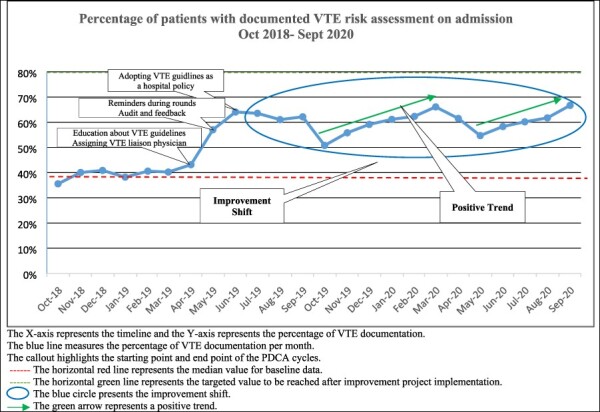
Run chart: VTE risk assessment documentation on admission over time.

**Figure 2:**
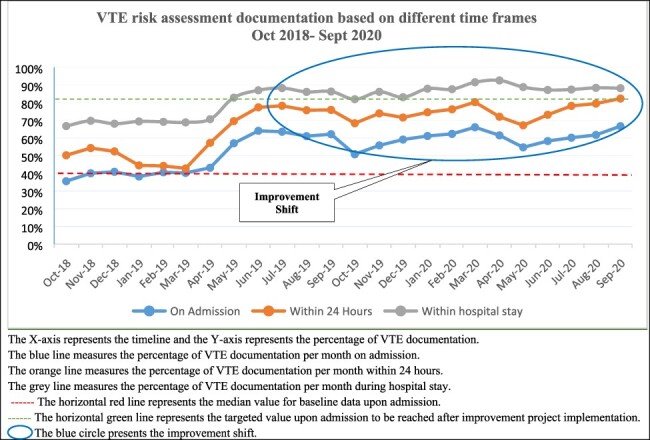
Run chart: VTE risk assessment documentation on admission, within 24 hours and during hospital stay.

Run charts presented the percentage of documented VTE risk assessment per month over the period October 2018–September 2020 (Figs. 1 & 2). The presented data showed some evidence of improvement in the VTE risk documentation with PDCA Cycles implementation. There was an increase in VTE risk documentation upon admission (Fig. 1) following the month of May when a significant shift was identified with more than six consecutive data points above the mean value presented as the red line. The percentage documentation reached 64% in June, and the results were sustained above the baseline median (39%) for 15 months after the quality improvement project implementation. The improvement shift was highlighted in a blue circle in Fig. 1. Furthermore, positive trends were identified in October 2019 till March 2020 and in May–September 2020, the compliance went down for a 2-month period between the two trends coinciding with the spike of COVID cases at the hospital.

On the other hand, there was an improvement in VTE risk documentation within 24 hours. The percentage of documentation increased from 44% in March 2019 before quality improvement implementation and reached 82% in September 2020. In addition, the overall documentation of VTE documentation within hospital stay, regardless of the completion timeframe constraint, increased from 69% in March 2019 before quality improvement implementation and reached 88% in September 2020. A positive improvement shift was evident and presented in Fig. 2.

### VTE incidences rate due to hospitalization

There was little evidence of a reduction in the VTE incidences rate following the implementation of the quality improvement project. It decreased from 0.5% (*n* = 44) pre-intervention to 0.4% (*n* = 35) post-intervention (relative decrease of 1%); this decrease was not statistically significant (*P*-value <0.397) and might be due to chance.

## Discussion

### Statement of principal findings

It has been shown in this study that a sustainable increase in VTE risk assessment documentation upon admission can be achieved by implementing a quality improvement framework. Following the FOCUS PDCA methodology, the improvement team implemented multicomponent interventions, which included educational sessions targeting physicians, sharing VTE documentation results with physicians, and daily reminders during rounds. Assigning a VTE liaison physician within each clinical specialty and updating and communicating the hospital adopted VTE guidelines were essential for improvement.

The results, including 17 612 records of patients admitted to the hospital during the study period (October 2018–December 2019), showed a significant increase in the percentage of patients who were risk assessed for VTE after quality improvement interventions were implemented. The percentage of patients assessed for VTE risk upon admission had increased from 42% at baseline data (pre-intervention) to 60% after project implementation (post-intervention). There were no major changes in the characteristics of patients admitted to the target hospital before and after the implementation of the quality improvement team likely to influence the observed changes in the documentation of VTE risk assessment in the patient electronic medical records. Moreover, the percentage of patients assessed for VTE risk within 24 hours and during hospital stay regardless of time constraint had increased to 76% and 88%, respectively. Improvement shift and trends were observed after quality improvement project implementation and improvement was sustained for 15 months.

However, the project results did not reach the goal of 80% VTE risk assessment documentation upon admission as set by the improvement team and there was no significant impact on the patient safety by demonstrating only a slight non-significant decrease in the VTE incidences rate. It is essential to highlight that the healthcare system is a complex social system where improving care through changing the behaviour of healthcare providers requires complex intervention. As described by the UK Medical Research Council (MRC) framework, complex interventions are interventions with a number of interacting components, including behavioural characteristics and the context of delivering those behaviours [20]. Understanding behavioural and environmental factors from both intervention providers and recipient’s perspectives are crucial to developing an effective intervention. This would support explaining why the VTE risk assessment documentation during the hospital stay, regardless of time constraint, reached 88% while on admission, reached only 64%; there are other barriers and facilitators that need to be further investigated following a theoretical framework to inform future interventions.

The implemented interventions, including education, assigning liaison physicians, reminders during rounds, audits, and feedback, will likely be transferable to other implementation studies in similar hospital settings, while taking into consideration the barriers and facilitators specific to them.

### Interpretation within the context of the wider literature

The findings from this project align with what has been reported in the literature that using the PDCA quality improvement methodology would improve clinical practice [21]. Moreover, it was reported that few of the PDCA quality improvement projects did reach their set objectives [21]. The multi-component interventions used in the project, such as education, audit and feedback, reminders, had proven to increase the implementation of the VTE clinical practice guidelines [15, 22].

### Interpretation for policy, practice, and research

Exploring the healthcare problem in-depth, the perceptions, preferences, and needs of those delivering and receiving the intervention are highly fundamental to identifying the determinants of behaviour change to inform effective interventions [23, 24]. Furthermore, identifying and developing a framework or theory to inform the intervention, such as behaviour change, would support the intervention effectiveness [25, 26]. The theory supports the translation of the identified behaviour change determinants into practical intervention components [23, 24, 27] and covers what worked and under which context to inform decision-making [28]. Throughout the intervention development process, healthcare providers should be involved through the iterative cycle of developing the intervention and using quantitative and qualitative research methods to support measuring processes and outcomes of the intervention [27]. The interventions implemented in future quality improvement projects could follow a formal framework to explain the problem and inform the interventions to support identifying the success factors within the interventions [29, 30].

### Strengths and limitations

At the study level, SQUIRE guidelines were followed to evaluate the implementation of the quality improvement project [12]. The use of secondary, routinely collected clinical data was one of the main strengths during the implementation of this project. The data analysis included 17 612 electronic records of patients admitted to the hospital during the study period. Moreover, continuous data collection was conducted over time, and improvement progress was reported and discussed. The sustainability of the results, including a positive trend during the 15 months after the quality improvement project implementation, can probably be attributed to the close monitoring and reporting of the data to the concerned staff on a regular basis. The interventions involved several clinical specialities formally attached to the project by assigning a VTE champion from each speciality. Targeted communication during daily rounds to physicians about completing the VTE risk assessment might explain the maintenance of results in a positive trend.

The intervention period was 3 months, which limited the number of activities that were done before post-intervention outcome data were collected. The interventions were based on the best practices in the medical field rather than a theoretical model. Other limitations were observed in this project: the improvement team did not investigate the potential barriers to the project’s success and did not use iterative PDCA cycles; thus, there was no link between the knowledge gained from one PDCA cycle to the next [30]. The lessons from one cycle did not inform the next since all cycles were implemented in parallel as per the initial plan. Thus, the effectiveness of each intervention was not measured separately. It is not easy to know what worked and for whom. Also, following iterative cycles of the intervention would have facilitated getting feedback from the involved healthcare providers in implementing the potential solutions and assessing their acceptability until the desired change is achieved [27]. The outcome indicator, VTE incidences, needs to be monitored over a more extended period for a meaningful evaluation of VTE interventions.

## Conclusion

This study was a process evaluation conducted within an acute care hospital in the field of quality improvement. It was found that the percentage of patients who were risk assessed for VTE was significantly increased after quality improvement interventions were implemented in a hospital setting. Moreover, evaluating implementation in the context of current practice provided a valuable insight into the general challenges faced when implementing the FOCUS PDCA methodology. Further, complex interventions such as implementation interventions need to be analysed to understand better the linkages between the active components of action and the impact on outcomes. This improvement project will likely be theoretically transferable to other implementation studies in similar hospital settings, mainly when considered alongside the use of theory to link the observed change to each intervention individually for successful implementation and its evaluation.

## Supplementary Material

mzae019_Supp

## Data Availability

All relevant data have been presented within the manuscript.
